# Histological Evaluation of the Healing Process of Various Bone Graft Materials after Engraftment into the Human Body

**DOI:** 10.3390/ma11050714

**Published:** 2018-05-02

**Authors:** Sang Hyun Jo, Young-Kyun Kim, Yong-Hoon Choi

**Affiliations:** 1 School of Dentistry, Seoul National University, 101 Daehak-ro, Jongro-gu, Seoul 03080, Korea; cerebral@snu.ac.kr; 2 Department of Oral and Maxillofacial Surgery, Section of Dentistry, Seoul National University Bundang Hospital, 82, Gumi-ro 173 Beon-gil, Bundang-gu, Seongnam city 13620, Gyeonggi-do, Korea; 3 Department of Dentistry & Dental Research Institute, School of Dentistry, Seoul National University, Seoul 03080, Korea; 4 Department of Conservative Dentistry, Section of Dentistry, Seoul National University Bundang Hospital, 82, Gumi-ro 173 Beon-gil, Bundang-gu, Seongnam city 13620, Gyeonggi-do, Korea

**Keywords:** autogenous tooth bone graft, AutoBT^®^, allograft, DBX^®^, xenograft, Bio-Oss^®^, new bone formation

## Abstract

The purpose of this study was to measure the level of new bone formation induced by various bone graft materials to provide clinicians with more choices. The samples were divided into three groups: group 1 (*n* = 9: allograft + xenograft, DBX^®^, San Francisco, CA, USA + Bio-Oss^®^, Princeton, NJ, USA), group 2 (*n* = 10: xenograft, Bio-Oss^®^), and group 3 (*n* = 8: autogenous tooth bone graft, AutoBT^®^, Korea Tooth Bank, Seoul, Korea). The average duration of evaluation was 9.56, 2.50, and 3.38 months, respectively. A tissue sample was taken from 27 patients during the second implant surgery. New bone formation was measured via histomorphometry, using a charge-coupled device camera, adaptor, and image analysis software. Total bone area, total area, and ((total bone area/total area) × 100) was measured to determine the extent of new bone formation. The mean value of the total bone area was 152,232.63 μm^2^; the mean value of the total area was 1,153,696.46 μm^2^; and the mean total bone area/total area ratio was 13.50%. In each comparison, there was no significant difference among the groups; no inflammation or complications were found in any of the groups. AutoBT^®^, an autogenous tooth bone graft, resulted in a level of bone formation similar to that using allografts and xenografts.

## 1. Introduction

When there is insufficient residual bone, implant placement becomes difficult. A deficiency of alveolar bone and pneumatization of the maxillary sinus causes bone loss. If an operator places an implant in this state, the bone will not cover the surface of the implant and might not result in a therapeutic effect [1]. Lekholm et al. suggested that >1 mm of buccal and lingual bone is needed on the implant surface. To increase the amount of available bone, various bone graft materials are used, including autogenous bone, allogeneic bone, xenograft, and synthetic bone [2].

Although autogenous bone is the ideal graft material, it has been suggested that donor site problems and limitations of harvesting amount are significant disadvantages. Therefore, development of allografts, xenografts, synthetic bone, etc., that can replace autogenous bone is ongoing, and studies on biodegradable graft material using tissue engineering techniques have been conducted [3]. However, autogenous bone is experiencing a resurgence in use because of recent developments in technology, such that autogenous bone is said to be the ideal implant. Recently, the use of autogenous tooth bone grafts has been increasing, and has been accomplished by extracting, processing, and grafting autogenous teeth. Importantly, autogenous tooth bone grafts are absorbed slowly by bone at the site of implantation and can form lamellar bone [4,5].

Allogeneic bone is made by processing cortical bone and cancellous bone obtained from a deceased donor, which can lead to immune rejection; however, allogeneic bone has superior osteoconduction and osteoinduction abilities. In the case of xenografts, bones of other animals (e.g., cattle and pigs) are collected and used; immune reactions can occur and transmission of viruses is possible, but these occur at a very low rate with the development of new technology. Synthetic bone can be used to produce new bone through a combination of hydroxyapatite and beta-tricalcium phosphate.

In the case of autogenous tooth bone graft material, the extracted tooth is made as a graft and transplanted into a tooth donor; it has been reported to be replaced with new bone during the healing process and histological observations show an abundant amount of lamellar bone. The brand name of autogenous tooth bone graft material is AutoBT^®^ (Korea Tooth Bank, Seoul, Korea). It has also been reported that robust bone remodeling is observed, and excellent osteoinduction and osteoconduction are observed during bone healing. However, AutoBT^®^ differs from autogenous bone in that it has no osteogenic effect. Approximately 90% of the organic material present in the dentin consists of collagen fibers, primarily type I collagen, and these fibers play an important role in calcification. The remaining organic components consist of non-collagenous proteins, carbohydrate, lipid, citrate, lactate, etc. The autogenous tooth bone graft material induces active new bone formation by osteoinduction and is gradually resorbed. With time, new bone is remodeled into a more stable bone structure, resulting in noticeable trabecular bone formation [4,5,6].

Osteogenesis, osteoinduction, and osteoconduction are the major mechanisms involved in new bone formation. Osteogenesis occurs in autografts and provides scaffolds, growth factors for osteogenesis, and cells that produce bone. Osteoinduction occurs in autogenous bone, allogeneic bone, and xenograft; this is a bone regeneration mechanism that induces osteoclast differentiation into bone cells in response to bone morphogenetic protein (BMP) or other growth factors. Osteoconduction occurs in autogenous bone, allogeneic bone, xenograft, and synthetic bone; it serves as a scaffold base and plays a role in bone regeneration. When the role of the scaffold is completed, this form of the bone is absorbed and replaced with new bone [7].

Histological evaluation of the bone healing response following transplantation of various bone graft materials into the human body facilitates the surgeon’s use of bone graft material and sets the healing period of the implant, in accordance with the patient’s clinical situation. Thus, we conducted this study to histologically evaluate the healing process of three types of bone graft materials.

## 2. Materials and Methods

Tissue samples were collected from 27 patients who underwent implant placement with guided bone regeneration; samples were divided into allograft + xenograft, xenograft, and autogenous tooth bone graft groups, and graft material was placed. The number of samples in each group is shown in Table 1. The mean duration of the observation period after placement of graft materials was 9.56 months in Group 1 (allograft + xenograft (DBX^®^ + Bio-Oss^®^)), 2.50 months in Group 2 (xenograft (Bio-Oss^®^)), and 3.38 months in Group 3 (autogenous tooth bone graft (AutoBT^®^)). The mean age of patients in Group 1 was 59.6 years, ranging from 41 to 72 years, with four males and five females. The surgical implantation sites included one posterior mandibular and eight posterior maxillary teeth. The mean group 2 patient age was 54.5 years, ranging from 28 to 80 years, with four males and six females. The surgical sites included six posterior mandibular, one anterior maxillary, and three posterior maxillary teeth. The mean group three patient age was 55.5 years, ranging from 41 to 73 years, with five males and three females. The surgical sites included six posterior mandibular, one anterior maxillary, and one posterior maxillary teeth.

This study was conducted in accordance with the Declaration of Helsinki. Samples were collected with a #15 surgical blade during the second implant surgery. Tissue specimens were stored in 10% formalin solution and histological studies were performed after the approval of the Bioethics Review Committee of Seoul National University Bundang Hospital (IRB No. B-1102/122-802). The 27 specimens were demineralized in 10% formic acid for 14 days, then embedded in paraffin. Serial sections of 5-μm thickness were obtained through the horizontal plane of the vertical alveolar defects, from which two sections that contained the entire defect were selected and stained with hematoxylin and eosin. For histomorphometric analysis, an optical microscope (BX51, Olympus Co., Tokyo, Japan), connected to a computer and charge-coupled device (CCD) camera (SPOT Insight 2-megapixel Scientific CCD Digital Camera System, Diagnostic Instruments Inc., Sterling Heights, MI, USA), and an adaptor (U-CMA3, Olympus Co., Tokyo, Japan), were used to take images of the samples. Image analysis was performed with SPOT Software V4.0 (Diagnostic Instruments Inc., Burroughs, Sterling Heights, MI, USA). The total bone ratio was measured using Pro Plus^®^ (Media Cybernetics Inc., Warrendale, PA, USA) analysis software. The area was calculated by an examiner who was unaware of the type of grafted material, and was measured in µm for tissues at 200× magnification. Three graft materials were used: DBX^®^ (SYNTHES, Oberdorf, Switzerland), Bio-Oss^®^ (Geistlich Pharm AG, Wolhusen, Switzerland), and AutoBT^®^ (Korea Tooth Bank Co., Seoul, Korea).

The histomorphometric method is a measurement program that first measures the total bone area in tissue photographs, then measures only the area of new bone formation. Then, the new bone formation value is calculated for each tissue photograph as follows: ((total bone area/total area) × 100). Based on the derived values, the mean ± standard deviation of each group was obtained.

1. Total bone area (TB): original bone area + new bone formation area
2. Total area (T): existing bone area + new bone formation area + soft tissue
3. New bone formation area (TB/T): total bone area/total area ratio

Twenty-seven samples were evaluated; TB, T, and TB/T were investigated using histomorphological analysis. The overall mean value in each study was also determined. The Kruskal-Wallis test (*p*-value < 0.05) was used to evaluate the data, using SPSS (Ver. 12.0, Chicago, IL, USA).

In 2014, Kim studied the composition and nanoscale structure of various bone graft materials. A scanning electron microscope (SEM, S-4800, Hitachi, Tokyo, Japan) was used to examine the surface structure of a variety of bone graft materials. SEM revealed that the surface of AutoBT^®^ crown, AutoBT^®^ root, Bio-Oss^®^, and autogenous mandibular cortical bone was covered with a 7-nm-thick platinum (Pt) coating when the instrument was operated at 15 kV [8].

## 3. Results

**Figure 1:**
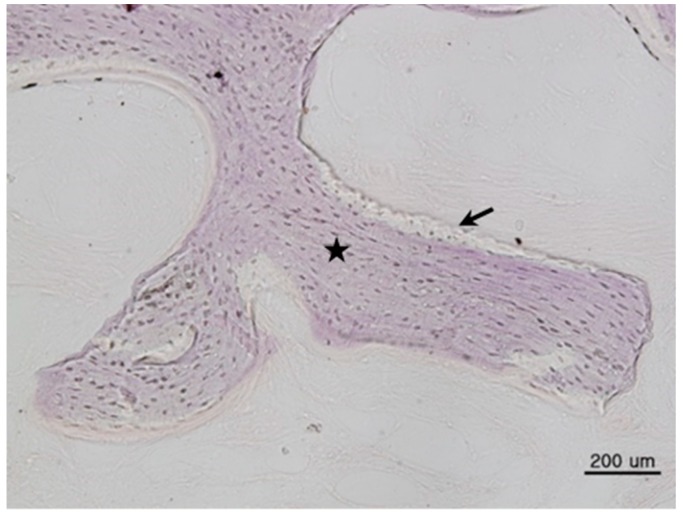
Histomorphometric image of Group 1 (DBX^®^ + Bio-Oss^®^). New bone (arrows) was surrounded by bone marrow (asterisks) (hematoxylin and eosin stain, 200×, scale bar = 200 µm).

**Figure 2:**
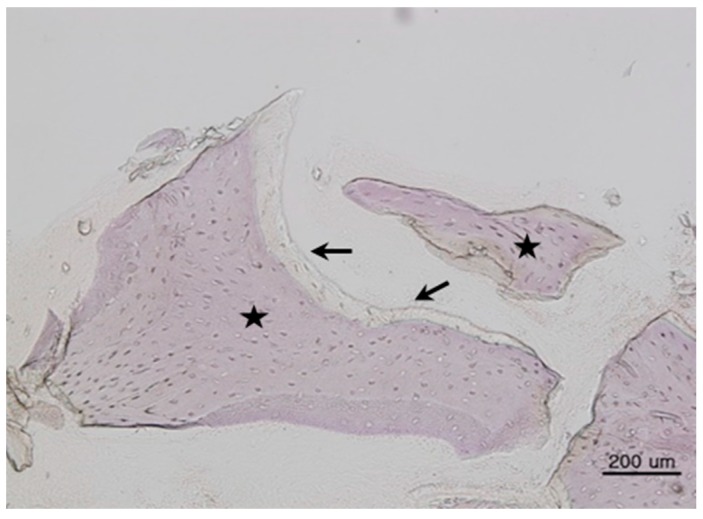
Histomorphometric image of Group 2 (Bio-Oss^®^). New bone (arrows) was surrounded by bone marrow (asterisks) (hematoxylin and eosin stain, 200×, scale bar = 200 µm).

**Figure 3:**
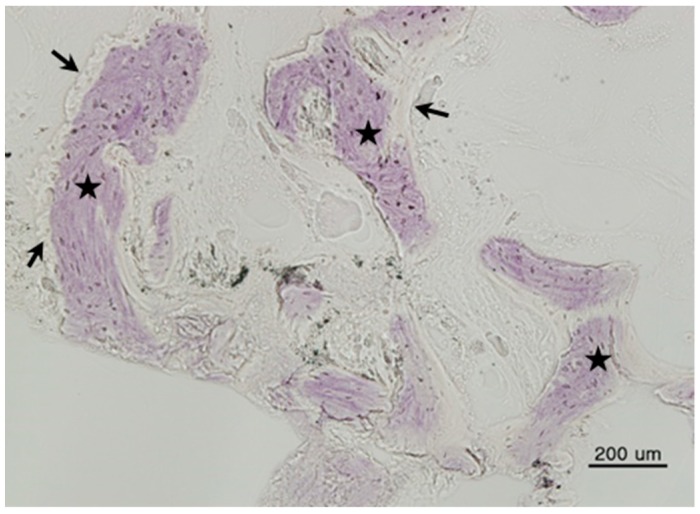
Histomorphometric image of Group 3 (AutoBT^®^ Block). New bone (arrows) was surrounded by bone marrow (asterisks) (hematoxylin and eosin stain, 200×, scale bar = 200 µm).

All bone grafts were successfully performed; no inflammation or complications were observed at the surgical site. Mature new bone was found surrounding each implant. Histologic images are shown in Figure 1, Figure 2 and Figure 3.

### 3.1. Total Bone Area (TB)

**Figure 4:**
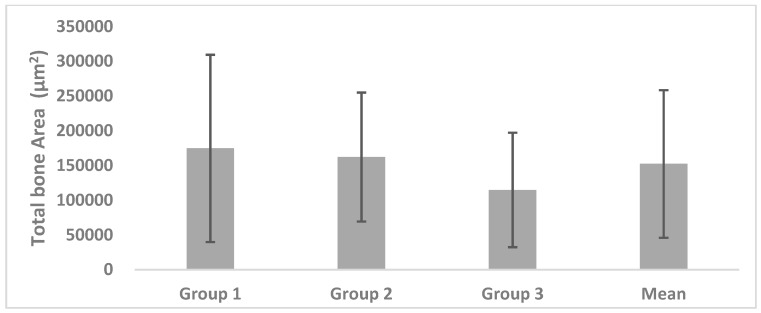
Histomorphometric quantification of the total bone area.

TB in Group 1 measured 174,534.66 ± 134,821.73 µm^2^, with 162,095.91 ± 92,843.26 µm^2^ in Group 2 and 114,813.76 ± 82,338.92 µm^2^ in Group 3; the mean value of the groups was 152,232.63 ± 106,238.70 µm^2^. There were no statistically significant differences among the three groups (Figure 4).

### 3.2. Total Area (T)

**Figure 5:**
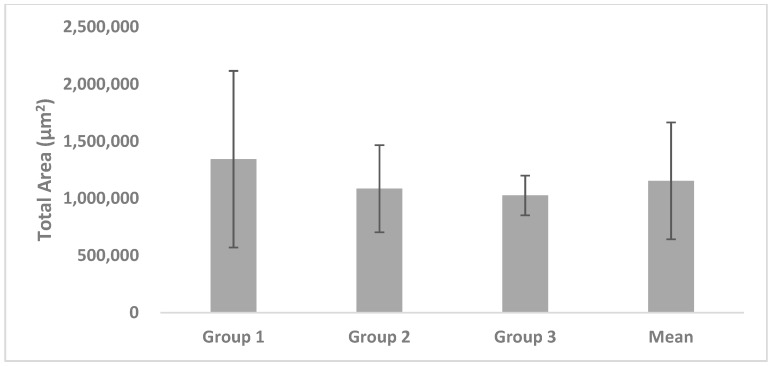
Histomorphometric quantification of the total area.

T of Group 1 measured 1,342,893.52 ± 772,620.49 µm^2^, with 1,085,757.52 ± 381,502.91 µm^2^ in Group 2, and 1,025,773.45 ± 173,489.18 µm^2^ in Group 3; the mean value of the three groups was 1,153,696.46 ± 511,233.81 µm^2^. There were no significant differences among the groups (Figure 5).

### 3.3. Total Bone Area/Total Area (TB/T)

**Figure 6:**
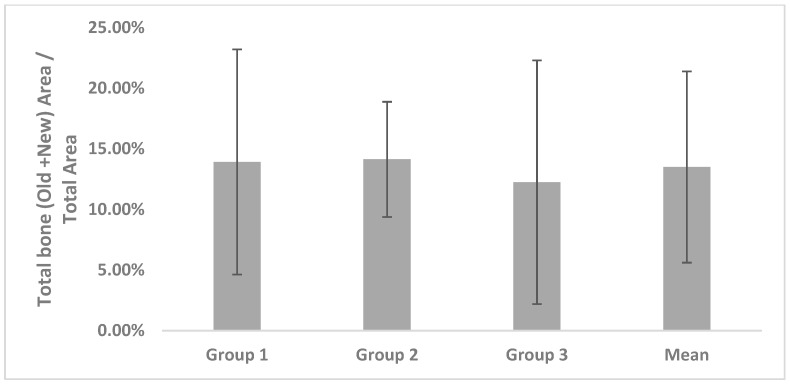
Ratio of total bone area/total area.

The total area was divided by the total bone area to measure the effectiveness of the grafted materials. TB/T in Group 1 was 13.92% ± 9.28%, with 14.13% ± 4.75% in Group 2 and 12.25% ± 10.05% in Group 3; the mean value of the three groups was 13.50% ± 7.89%. There were no significant differences among the groups (Figure 6).

### 3.4. Nanoscale Structure of Bone Graft Material (SEM)

**Figure 7:**
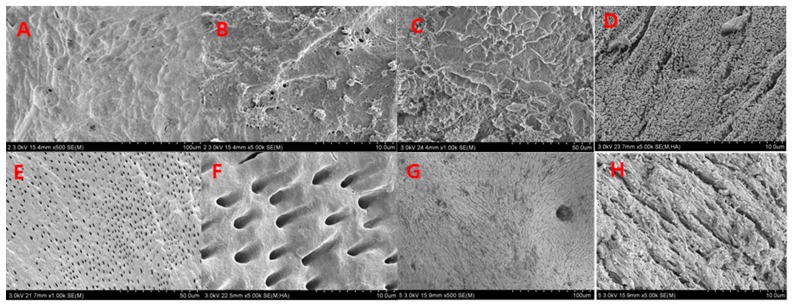
SEM views of the different types of bone graft materials. (**A**) Autogenous cortical bone (500×); (**B**) Autogenous cortical bone (5000×); (**C**) AutoBT crown (1000×); (**D**) AutoBT crown (5000×); (**E**) AutoBT root (1000×); (**F**) AutoBT root (5000×); (**G**) Bio-Oss (500×); and (**H**) Bio-Oss (5000×).

Natural autogenous mandibular cortical bone showed wave-shaped compact patterns. Under high magnification, irregular fiber patterns and a mixture of ribbon-shaped structures of varying sizes appear. AutoBT^®^ showed different structures in the crown and root portion. AutoBT^®^ crown surfaces of the sections were generally homogeneous, with small dentinal tubules of 1.0 to 1.5 µm in length. In comparison with AutoBT^®^ root sections, these showed a more compact pattern. Moreover, small particles that were speculated to be hydroxyapatite were observed between the dentinal tubules. The surfaces of AutoBT^®^ root were generally homogeneous, and minute cross-sections of the dentinal tubules were observed. In high-magnification photographs, the surfaces between the dentinal tubules present at 5–10-µm intervals showed slightly rough patterns. The section surfaces of the xenogeneic bone graft material Bio-Oss^®^ were less compact, whereas a regular arrangement of small tubules of 5 µm in width was observed [8] (Figure 7).

## 4. Discussion

Many studies have examined the healing process involving bone graft materials, but there are few studies on the healing process in biopsied human tissue. Although it is very difficult to conduct this research because it is limited by bioethical concerns, human studies provide the most useful information for the guidance of clinical treatment.

For guided bone regeneration, it is very important to study the alveolar bone, especially the marginal bone response around the implant. It has been shown that when bone grafting is performed around the implant and a load is applied, there is a high possibility of bone resorption around the implant when the prosthesis is initially attached [9,10]. Therefore, to evaluate the healing process at the alveolar bone surface after bone grafting, bone surface specimens were obtained with a surgical blade and the healing process was evaluated via histological methods. In this study, we investigated differences in graft materials regarding the formation of new bone during guided bone regeneration; we compared allogeneic bone, xenograft, and autogenous bone graft, all of which are widely used in clinical practice.

Allogeneic DBX^®^ is a bone graft material composed of demineralized freeze-dried bone allograft and hyaluronic acid, mixed at a ratio of 32:68 *w*/*w*%. DBX^®^ is an excellent healing material because of its osteoconduction and osteoinduction properties [11]. In addition, DBX^®^ has been reported to shorten the preparation time because it can be used without mixing with other bone graft materials [12]. In one study, the rates of new bone formation in autogenous bone, when using DBX^®^ and Bio-Oss^®^, were 51.0% ± 18.3% and 52.5% ± 10.7%, respectively. New bone formation was robust in all groups after four months of treatment [11]. However, our study suggests that the degree of new bone formation was very high because it was related to a sinus bone graft with an excellent healing process. In addition, the sinus cavity is a well-contained defect that is surrounded by walls; thus, any bone graft material may tend to demonstrate a good healing process. Some studies have shown that Bio-Oss^®^ molecules are similar in physical properties to human bone tissue. Notably, the Bio-Oss^®^ cancellous bone trabeculae create a conductive pathway in the new bone [13]. Many studies have shown successful results in new bone reconstruction. Lamellar bone/woven bone ratios and new bone/implant ratios have been measured to determine the suitability of the materials and the recovery of implant materials; notably, all showed good recovery [4]. Bio-Oss^®^ and Inducera^®^ (a low temperature-treated heterogeneous bone graft material) have been reported to contain a crystal structure similar to human bone tissue and to exhibit excellent bone conduction healing ability. Further, there have been reports of robust bony healing after 1–2.5 months of guided bone regeneration in heterogeneous bone [14]. Bone grafting with synthetic bone sinus lifting has been reported as reliable [13]. In a sinus bone graft study involving a combination of DBX^®^ and Bio-Oss^®^, the rate of new trabecular bone formation was approximately 7–17% [12]. Another study indicated that AutoBT^®^ and Bio-Oss^®^ both demonstrated favorable wound healing, implant stability, and new bone formation. The vertical dimensions of alveolar bone increased; moreover, implant stability quotients were similar in those two. Notably, the TB/T values of AutoBT^®^ and Bio-Oss^®^ were 31.24 ± 13.87% and 35.00% ± 19.33%, respectively [15]. These were higher than our study values; we suspect that this is because the investigators in that study utilized a longer follow-up time.

Demineralized dentin matrix (DDM) has been reported to perform well as a carrier of recombinant human BMP-2, as DDM provides robust bone formation via bone induction and bone conduction [16]. In that study, specimens using the powder and block forms of AutoBT^®^ as graft materials were collected 2–5 months after transplantation; quick fixation was performed. Autogenous DDM is designed to complement deficiencies in autogenous bone and other alternative bone, and exhibits robust osteoinduction and osteoconduction abilities. In addition, its histologic appearance in the recovery phase resembles autogenous bone [6,17,18,19].

There is a difference in bone composition according to the site of the tooth; further, the ratio of new bone/bone graft material was 21–94% [6]. Histopathological analysis has shown that in loose fibrous tissue with sufficient angiogenesis and bone formation, newly-formed osteoid replaces the molecules of reabsorbed AutoBT^®^ [11]. The results of that study also showed that osteoid and osteoblasts were clustered around AutoBT^®^, and that the contents of the organic matter in the crown and root were different; moreover, the collagen content was also different. This is thought to be due to the distribution of nutrient-diffusion during new bone formation [20].

In our study, the TB/T ratio of the alveolar bone surface was assessed; however, the duration of the healing was variable, making it difficult to compare directly with other studies. In this study, DBX^®^ + Bio-Oss^®^, Bio-Oss^®^, and AutoBT^®^ were all observed to form new bone. The mean values of TB did not significantly differ among each of the three groups.

In this study, both osteogenesis and healing patterns were similar on the surfaces of both Groups 1 and 3. There was no inflammation in the periphery, nor were other complications observed; new bone and new bone marrow were both formed. Notably, new bone formation was observed in almost all samples. This confirms that AutoBT^®^ exhibits results similar to those reported in other papers and that it has effects similar to other bone graft materials [6,18,20,21].

The total bone ratio was maintained at 12–14%, indicating that the degree of bone formation on the surface layer is insufficient and implying that many other tissues are included. Thus, there is a possibility that bone loss may be increased if an excessive load is placed on the implant or if an infection (e.g., peri-implantitis) occurs. Additionally, the variation of the total bone ratio may be increased because of interindividual differences.

There were some limitations in this study. First, this is a retrospective study that used stored specimens. The healing periods in each group were different and it was difficult to histologically distinguish between mature bone and immature bone. Thus, standardization of patients, as well as the age of the stored tissue, might be required to obtain more precise data. Second, the quantity of placed graft material should be standardized. Some patients may exhibit a much greater graft material/implant area ratio, such that the areas of new bone formation and residual graft material might not be standardized.

In a study of the nanoscale structure of each graft material, the density, roughness, and homogeneity of AutoBT^®^ were shown to be relatively similar to those of autogenous cortical bones. The irregular surface between dentinal tubules, as well as the circular- or ribbon-shaped structures of autogenous cortical bones, suggest the presence of both mineralized and organic materials. There was no statistical difference among the three groups in terms of TB, T, or TB/T ratio. The AutoBT^®^ demonstrates excellent osteoconduction and osteoinduction potential, and shows robust bone formation. If the quantity of organic matter can be adjusted to a certain proportion during the process of preparing the graft material, in order to provide a more homogeneous result, the osteoconduction and osteoinduction abilities can be further optimized; this improvement could be readily applied in patients.

## Figures and Tables

**Table:** 

Mean Value	Group 1	Group 2	Group 3	Mean
**Total bone area (µm^2^)**	174,534.66 ± 134,821.73	162,095.91 ± 92,843.26	114,813.76 ± 82,338.92	152,232.63 ± 106,238.70

**Table:** 

Mean Value	Group 1	Group 2	Group 3	Mean
**Total bone area (µm^2^)**	1,342,893.52 ± 772,620.49	1,085,757.52 ± 381,502.91	1,025,773.45 ± 173,489.18	1,153,696.46 ± 511,233.81

**Table:** 

Mean Value	Group 1	Group 2	Group 3	Mean
$\frac{\mathrm{Total}\;\mathrm{bone}\;\left(\mathrm{Old}+\mathrm{New}\right)\;\mathrm{area}}{\mathrm{Total}\;\mathrm{area}}$	13.92% ± 9.28%	14.13% ± 4.75%	12.25% ± 10.05%	13.50% ± 7.89%

**Table 1:** Distribution of subjects.

Group	Composition	Number of Samples	Average Duration (Months)
**1**	Allograft + Xenograft (DBX^®^ + Bio-Oss^®^)	9	9.56
**2**	Xenograft (Bio-Oss^®^)	10	2.50
**3**	Autogenous tooth bone graft (AutoBT^®^)	8	3.38

## References

[1] Park S.J., Seon H.G., Koh S.W., Chee Y.D. (2012). Retrospective clinical study on marginal bone loss of implants with guided bone regeneration. Maxillofac. Plast. Reconstr. Surg..

[2] Lekholm U., Adell R., Lindhe J., Branemark P., Eriksson B., Rockler B., LIindvall A., Yoneyama T. (1986). Marginal tissue reactions at osseointegrated titanium fixtures (II) A cross-sectional retrospective study. Int. J. Oral Maxillofac. Surg..

[3] Sheikh Z., Najeeb S., Khurshid Z., Verma V., Rashid J., Glogauer M. (2015). Biodegradable materials for bone repair and tissue engineering applications. Materials.

[4] Jeong H.R., Hwang J.H., Lee J.K. (2011). Effectiveness of autogenous tooth bone used as a graft material for regeneration of bone in miniature pig. J. Korean Assoc. Oral Maxillofac. Surg..

[5] Tazaki J., Murata M., Yuasa T., Akazawa T., Ito K., Hino J., Nida A., Arisue M., Shibata T. (2010). Autograft of human tooth and demineralized dentin matrices for bone augmentation. J. Ceram. Soc. Jpn..

[6] Kim Y.K., Kim S.G., Byeon J.H., Lee H.J., Um I.U., Lim S.C., Kim S.Y. (2010). Development of a novel bone grafting material using autogenous teeth. Oral Surg. Oral Med. Oral Pathol. Oral Radiol. Endod..

[7] Zwingenberger S., Nich C., Valladares R.D., Yao Z., Stiehler M., Goodman S.B. (2012). Recommendations and considerations for the use of biologics in orthopedic surgery. BioDrugs.

[8] Kim Y.K., Kim S.G., Yun P.Y., Yeo I.S., Jin S.C., Oh J.S., Kim H.J., Yu S.K., Lee S.Y., Kim J.S. (2014). Autogenous teeth used for bone grafting: A comparison with traditional grafting materials. Oral Surg. Oral Med. Oral Pathol. Oral Radiol..

[9] Lee J.Y., Lee J., Kim Y.K. (2013). Comparative analysis of guided bone regeneration using autogenous tooth bone graft material with and without resorbable membrane. J. Dent. Sci..

[10] Kim Y.K., Kim S.G., Lim S.C., Lee H.J., Yun P.H. (2010). A clinical study on bone formation using a demineralized bone matrix and resorbable membrane. Oral Surg. Oral Med. Oral Pathol. Oral Radiol. Endod..

[11] Lee K.J.H., Roper J.G., Wang J.C. (2005). Demineralized bone matrix and spinal arthrodesis. Spine J..

[12] Schwartz Z., Goldstein M., Raviv E., Hirsch A., Ranly D.M., Boyan B.D. (2007). Clinical evaluation of demineralized bone allograft in a hyaluronic acid carrier for sinus lift augmentation in humans: A computed tomography and histomorphometric study. Clin. Oral Implants Res..

[13] Maiorana C., Sigurta D., Mirandola A., Garlini G., Santoro F. (2006). Sinus elevation with alloplasts or xenogenic materials and implants: An up-to-4-year clinical and radiologic follow-up. Int. J. Oral Maxillofac. Implants.

[14] Kim Y.K. (2014). Bone graft using calcium phosphate dual-coating xenograft material (InduCera^®^): Case reports. J. Dent. Implant Res..

[15] Pang K.M., Um I.W., Kim Y.K., Woo J.M., Kim S.M., Lee J.H. (2017). Autogenous demineralized dentin matrix from extracted tooth for the augmentation of alveolar bone defect: A prospective randomized clinical trial in comparison with anorganic bovine bone. Clin. Oral Implants Res..

[16] Kim Y.K. (2014). Bone graft using two types of scaffolds and recombinant human bone morphogenetic protein-2: Case series study. Oral Biol. Res..

[17] Lee J.Y., Kim Y.K., Yi Y.J., Choi J.H. (2013). Clinical evaluation of ridge augmentation using autogenous tooth bone graft material: Case series study. J. Korean Assoc. Oral Maxillofac. Surg..

[18] Kim Y.K., Yun P.Y., Um I.W., Lee H.J., Yi Y.J., Bae J.H., Lee J. (2014). Alveolar ridge preservation of an extraction socket using autogenous tooth bone graft material for implant site development: Prospective case series. J. Adv. Prosthodont..

[19] Jun S.H., Ahn J.S., Lee J.I., Ahn K.J., Yun P.Y., Kim Y.K. (2014). A prospective study on the effectiveness of newly developed autogenous tooth bone graft material for sinus bone graft procedure. J. Adv. Prosthodont..

[20] Kim S.Y., Kim Y.K., Park Y.H., Park J.C., Ku J.K., Um I.W., Kim J.Y. (2017). Evaluation of the healing potential of demineralized dentin matrix fixed with recombinant human bone morphogenetic protein-2 in bone grafts. Materials.

[21] Jeong K.I., Kim S.G., Kim Y.K., Oh J.S., Jeong M.A., Park J.J. (2011). Clinical study of graft materials using autogenous teeth in maxillary sinus augmentation. Implant Dent..

